# Rumen-Protected Methionine Supplementation in the Diet Improved the Production Performance of Dairy Goats by Optimizing the Amino Acid Profile and Lipid Metabolism and Modulating the Colonic Microbiome

**DOI:** 10.3390/ani15233386

**Published:** 2025-11-24

**Authors:** Xingwei Jiang, Jiarui Wang, Yuhao Zhang, Jing Li, Huifeng Liu, Shengru Wu, Junhu Yao

**Affiliations:** 1 College of Animal Science and Technology, Northwest A&F University, Yangling, Xianyang 712100, China; jiangxingwei0115@163.com (X.J.);; 2 Key Laboratory of Livestock Biology, Northwest A&F University, Yangling, Xianyang 712100, China

**Keywords:** dairy goat, rumen-protected methionine, colonic microbiome, milk production

## Abstract

This study aimed to investigate the effects of rumen-protected methionine (RPM) on the production performance of lactating dairy goats. Thirty first-time lactating Guanzhong dairy goats with identical kidding dates and comparable body weights (41.17 ± 3.05 kg) were randomly assigned to two groups: (1) CON: basal diet and (2) RPM: basal diet + 7.5 g/day RPM. The duration of the experiment was 21 days. Compared with the CON group, the RPM group presented a significant increase in milk yield, 4% fat-corrected milk (FCM), and feed efficiency; however, no significant difference was observed in dry feed intake (DMI). Moreover, milk fat, protein, lactose, and SNF production was greater in the PRM group than in the CON group. Compared with the CON group, the RPM group presented higher nonesterified fatty acid (NEFA) and very-low-density lipoprotein (VLDL) levels, and no significant differences in the other metabolites were detected. The concentrations of acetate, propionate, and total volatile fatty acids (TVFAs) in the feces of the RPM group were significantly greater than those in the CON group; however, no significant differences were detected in the concentrations of isobutyrate, butyrate, and valerate. Furthermore, genera such as *Muribaculaceae*, *Bifidobacterium*, and *Christensenellaceae* were significantly enriched in the feces of the RPM group. Concurrently, Kyoto Encyclopedia of Genes and Genomes (KEGG) pathway analysis revealed that the RPM group was significantly enriched in pathways associated with amino acid synthesis, the immune system, and energy metabolism. In summary, dietary supplementation with RPM improved the lipid metabolism function of the liver, increased the abundance of beneficial bacteria such as *Muribaculaceae* and *Bifidobacterium* in the colon, and enriched microbial functions related to energy and amino acid metabolism, thereby enhancing colon fermentation and host metabolic status, ultimately improving the production performance of lactating dairy goats. These findings elucidate the positive effects of RPM on the production performance and metabolic health of dairy goats, potentially offering new perspectives and strategies for optimizing dairy production.

## Simple Summary

As living standards and consumption rise, demand for goat milk is increasing, making improvements in dairy goat feed efficiency and health a research priority. This study investigated the effects of supplementing rumen-protected methionine (RPM) on production performance and metabolic health in dairy goats. Thirty lactating goats were divided into two groups: one received a basal diet, and the other received the same diet supplemented with 7.5 g/d of RPM. Results showed that RPM significantly increased milk yield, fat-corrected milk, and feed efficiency without altering dry matter intake. Milk fat, protein, and lactose levels also increased. Elevated serum non-esterified fatty acids and very-low-density lipoproteins indicated enhanced lipid metabolism. Fecal microbiota analysis revealed higher abundances of beneficial bacteria such as *Muribaculaceae* and *Bifidobacterium*, along with enrichment of pathways related to amino acid and energy metabolism. These findings suggest that RPM improves metabolic health and production efficiency by optimizing intestinal lipid metabolism and microbial function. This study offers valuable insights for enhancing dairy goat productivity through nutritional strategies.

## 1. Introduction

The weeks following kidding represent a period characterized by heightened nutritional requirements and significant physiological changes for ruminants [1]. The perinatal period is defined as the three weeks preceding kidding and the three weeks following kidding. If the transition does not proceed smoothly, it is often associated with a decline in production performance and an increased incidence of disease [2]. In particular, a smooth and healthy transition during the first three weeks post- kid is critical for establishing high milk production, restoring body condition, and enhancing reproductive performance [3,4]. Most ruminants experience a deficiency in protein and energy during the initial weeks following kidding and utilize their body reserves to mitigate this adverse effect [5]. The breakdown of fat results in the release of nonesterified fatty acids (NEFAs) into the bloodstream, which serve as energy substitutes when the glucose supply is insufficient. However, in the early stages following kidding, excessive fat mobilization increases the flow of NEFAs to the liver, exceeding the capacity of liver cells to oxidize and utilize these fatty acids [6,7]. Excess NEFAs are re-esterified and stored in the liver as triglycerides (TGs), which may contribute to lipid deposition in the liver. In dairy ruminants, excessive accumulation of TG in the liver is linked to a decline in milk production and overall health [8,9]. Therefore, regulating and improving lipid metabolism should be considered an effective strategy for enhancing the production performance of ruminants.

Methionine (Met) is considered one of the most important limiting amino acids for ruminants, and dietary supplementation with Met has been proven to improve animal health and production performance [10]. Research has indicated that appropriate Met supplementation during the perinatal period can effectively mitigate the negative energy and protein balance during this phase [11,12,13,14]. Moreover, dietary supplementation with Met can promote complete hepatic fatty acid oxidation and gluconeogenesis, thereby increasing the body’s energy supply [15]. Previous studies have shown that supplementation with Met can improve one-carbon metabolism by providing the methyl groups required for DNA and protein methylation, thereby alleviating the adverse effects of nutritional deficiencies during the periparturient period [16,17,18]. Furthermore, Met can be converted into active methyl groups and S-adenosylmethionine (SAM) in the body and subsequently transformed into very-low-density lipoprotein (VLDL) and DNA methylation products [19]. In dairy cows, the use of various sources of rumen-protected methionine (RPM) (13.4 to 22.1 g/d) tends to increase milk yield, significantly enhancing protein and fat contents, as well as milk protein yield [20,21,22]. In dairy goats, supplementation with RPM (1–5 g/d) improved milk yield and protein and fat contents when fed at 2.5 g/d during mid-lactation [23]. However, results were inconsistent at lower crude protein (CP) diets (0–3 g/d, 10% CP) [24] and at higher Met doses (5 g/d [23]; 4 g/d (14–16% CP) [25]). Nonetheless, Boutinaud et al. [26] demonstrated that feeding RPM at adequate energy levels increased casein gene expression and reduced mammary cell apoptosis in lactating dairy goats. Based on the supplementation levels of RPM used in the aforementioned studies and the manufacturer’s recommendations for RPM, we selected a daily dose of 7.5 g RPM per dairy goat in the experimental diet. Although Met has been shown to positively affect production performance and health in dairy cows, there are currently few studies on Met supplementation in dairy goats, and no consensus exists regarding the production and metabolic responses to Met supplementation in this species.

Rumen-undegradable amino acids, rumen microbial protein, and rumen-protected amino acids are the primary sources of amino acids in the intestine of ruminants [27], and microbial metabolism plays a crucial role in protein metabolism [28]. Rumen-protected amino acids can bypass the rumen and reach the small intestine, thereby increasing the efficiency of amino acid utilization. When rumen-protected amino acids are supplemented in the diet, the importance of the gut microbiome, particularly in the colon, should be emphasized [29,30]. The colonic microbiota can metabolize dietary protein and nonprotein nitrogen in ruminants, thereby influencing nitrogen use efficiency [31]. Previous studies have shown that supplementing RPM in kid diets enhances the biosynthesis of butyrate and amino acids by the colonic microbiota, thereby improving growth performance, which is associated with the enrichment of beneficial bacteria in the colon (e.g., *Akkermansia_muciniphila* and *RUG099* spp.) [32]. However, the role of the colon microbiota in maintaining amino acid homeostasis and fermentation remains poorly understood, particularly in dairy ruminants, and warrants further investigation.

Therefore, we hypothesize that supplementing protected RPM in the late perinatal period can alleviate fat mobilization and the negative balance of energy and protein after kidding, thereby improving the production performance of dairy goats. This study aimed to investigate the effects of dietary supplementation with RPM on lactation performance, lipid metabolism, the blood amino acid profile, and the microbiota and function of the colon in dairy goats during the perinatal period.

## 2. Materials and Methods

### 2.1. Animals, Diets, and Management

Thirty primiparous Guanzhong dairy goats in early lactation, with the same kidding date and similar body weights (41.17 ± 3.05 kg), were randomly divided into two groups, housed individually in pens. The dairy goats belong to the Animal Husbandry Teaching and Experiment Base of the College of Animal Science and Technology, Northwest A&F University, and have been approved for use. The goats commenced the experiment immediately after parturition. All kids received colostrum at birth and were subsequently transferred to the kid house for standardized artificial lactation. The treatments consisted of the following: (1) the CON group (*n* = 15) received a basal diet without Met, and (2) the RPM group (*n* = 15) received a basal diet supplemented with 7.5 g/d RPM. The RPM supplement, procured from Hangzhou King Technology Feed Co., Ltd. (Hangzhou, China), contained 80% Met. The ruminal undegradability and small intestinal absorptive capacity of RPM were reported to be 83% and 98%, respectively. Consequently, the goats in the RPM group received an approximate delivery of 5.0 g/d of Met into the small intestine. The basal diet was formulated according to the recommendations of the NRC [33], and the ingredients and nutrient composition are presented in Table 1. Goats were fed twice daily at 06:30 and 17:30 h at 120% of the average weekly dry matter intake (DMI) to ensure that 5–10% of feed remained for accurate measurement of DMI. Milk was collected twice daily at 06:00 and 17:00 h. RPM was top-dressed onto the experimental diet and fed twice daily in equal amounts. The pens were equipped with automatic drinking water dispensers. The experimental period lasted from parturition until 21 days post-partum. The fecal and blood samples were collected 2 h after the morning feeding on the day 21.

### 2.2. Experimental Procedures and Sample Collection

Fresh feed and refused feed were collected weekly and subsequently stored at −20 °C for further analysis. The goats were weighed before morning feeding on days 0 and 21, and their average daily gain (ADG) was calculated over this period. The milk yield was recorded over three consecutive days when the goats were in milk for 19, 20 and 21 days. Milk samples (*n* = 15) were collected for three consecutive days on days 19, 20, and 21, maintaining an early-to-late sampling ratio of 3:2. The samples were stored in 50 mL centrifuge tubes at 4 °C for milk composition analysis. 4% fat-corrected milk (FCM) was calculated using the formula:

FCM = 0.4 × M + 15M × F

where M represents milk yield and F is the milk fat percentage. Feed efficiency was calculated as FCM divided by DMI. Blood samples (*n* = 15) were collected via the jugular vein at 21 days post-partum and two hours after morning feeding (centrifuged at 1000× *g* for 15 min). The serum was aliquoted and subsequently stored at −20 °C for further analysis. Two hours after the morning feeding, fecal samples (*n* = 15) were collected via rectal sampling and cryopreserved in liquid nitrogen for subsequent analysis.

### 2.3. Laboratory Analyses

The dry matter (DM, method 930.15), crude protein (CP, method 98.13), and acid detergent insoluble ash (ADIA, method 935.29) contents of the feed samples were analysed in accordance with the AOAC methods (2007). Neutral detergent fibre (NDF) and acid detergent fibre (ADF) were analysed according to the methods of Van Soest et al. [34], employing heat-stable α-amylase and expressed without residual ash. The starch content was quantified via anthrone colorimetry following acid hydrolysis via a commercial starch kit (Nanjing Jiancheng Bioengineering Institute, Nanjing, China).

The composition of goat milk, including fat, protein, lactose, solid nonfat (SNF), and milk urea nitrogen (MUN), was measured via infrared spectroscopy (MilkScan model FT+ 600, Foss Inc., Hillerød, Denmark). Blood samples were analysed for concentrations of nonesterified fatty acids (NEFA, A042-2), total bilirubin (TBIL, C019-1-1), albumin (ALB, A028-1-1), cholesterol (CHOL, A111-1-1), and triglycerides (TG, A110-1-1) via commercial kits (Jiancheng Bioengineering Institute, Nanjing, China) via enzymatic colorimetric methods. The concentrations of glucose (GLU), total protein (TP), blood urea nitrogen (BUN), high-density lipoprotein cholesterol (HDL), low-density lipoprotein cholesterol (LDL), and globulin (GLO) in the blood samples were determined via an automatic biochemical analyser (Hitachi 7600, Hitachi Group, Tokyo, Japan). The concentrations of very-low-density lipoprotein (VLDL), β-hydroxybutyric acid (BHBA), alanine aminotransferase (ALT), and aspartate aminotransferase (AST) were determined via an ELISA kit (Aoqing Biotech Company, Nanjing, China). The blood amino acid content was analysed via an automated amino acid analyser (JIC-5/V, Nippon Electron, Co., Ltd., Tokyo, Japan).

Fecal volatile fatty acids (VFAs) were analysed via gas chromatography (Agilent Technologies 7820A GC system, Santa Clara, CA, USA) via a capillary column (AT-FFAP: 30 m × 0.32 mm × 0.5 μm; ATECH Technologies Co., Ltd., Shanghai, China), following the removal of solid particles and protein from the sample as described previously by Li et al. [35]. Briefly, the initial temperature of the column oven was set at 90 °C, then increased to 120 °C at a rate of 10 °C/min and maintained for 3 min, followed by a further increase to 180 °C at the same rate, which was held for 5 min.

### 2.4. Fecal Microbiota 16S rRNA Gene Sequencing

Total genomic DNA extraction from the fecal microbial communities was performed in accordance with the instructions of the E.Z.N.A.^®^ soil DNA kit (Omega Biotek, Norcross, GA, USA), after which the quality, concentration and purity of the extracted DNA were determined.

Taking the extracted DNA as the template, the V3-V4 variable region of the 16S rRNA gene was amplified via PCR via the primers 5′-ACTCCTACGGGAGGCAGCAG-3′ and 5′-GGACTACHVGGGTWTCTAAT-3′, and the PCR products were recovered via 2% agarose gel electrophoresis. The recovered products were purified and quantified via a PCR Clean-up Kit (YuHua, Shanghai, China) and a Qubit 4.0 (Thermo Fisher Scientific, Waltham, MA, USA). The purified PCR products were built into libraries via the NEXTFLEX/Rapid DNA-Seq Kit. Sequencing was performed via the Illumina Nextseq2000 platform.

Using fastp [36] (https://github.com/OpenGene/fastp (accessed on 10 August 2025), version 0.19.6) software, quality control was carried out on the double-end original sequencing sequence, and FLASH [37] (version 1.2.11) software was used for mosaic. On the basis of default parameters, the DADA2 [38] plugin in the QIIME 2 process [39] is used to perform noise reduction processing on the optimized sequence after quality control stitching. To minimize the impact of sequencing depth on the subsequent alpha diversity and beta diversity data analysis, the number of all sample sequences was flattened. On the basis of the Sliva 16S rRNA gene database (v 138), taxonomic analysis of ASVs was performed via the naive bays classifier in QIIME 2. The 16S function prediction analysis was conducted via PICRUSt2 [40] (version 2.2.0) software. All the data are analysed in the auspicious cloud platform (https://cloud.majorbio.com (accessed on 28 August 2025)), and chiplot visualization mapping is then performed (https://www.chiplot.online/ (accessed on 28 August 2025)).

### 2.5. Statistical Analyses

To ensure adequate statistical power to detect the anticipated effect, we conducted an a priori power analysis. Based on the mean and standard deviation of milk yield (1.46 ± 0.48 vs. 2.13 ± 0.44) from a pilot study in dairy goats, a two-sided test was used with a significance level (α) set at 0.05 and a target statistical power of 80%. Sample size calculation was performed using the pwrss: Statistical Power and Sample Size Calculation Tools. R package version 0.3.1 (https://CRAN.R-project.org/package=pwrss (accessed on 10 September 2025)). The results indicated that the minimum required sample size per group was 9. In this study, the sample size per group (*n* = 13) exceeds the minimum required sample size (*n* = 9), ensuring a probability of over 80% to detect the specified effect size at the α = 0.05 significance level.

Statistical analysis was conducted via independent sample t tests and Kruskal-Wallis tests in SPSS version 23 (SPSS, Inc., Chicago, IL, USA). The data are presented as the means ± standard errors (SEs). Statistical significance was defined as *p* < 0.05, with *p* < 0.01 considered highly significant, and 0.05 ≤ *p* < 0.10 regarded as a tendency.

## 3. Results

### 3.1. Production Performance

**Figure 1:**
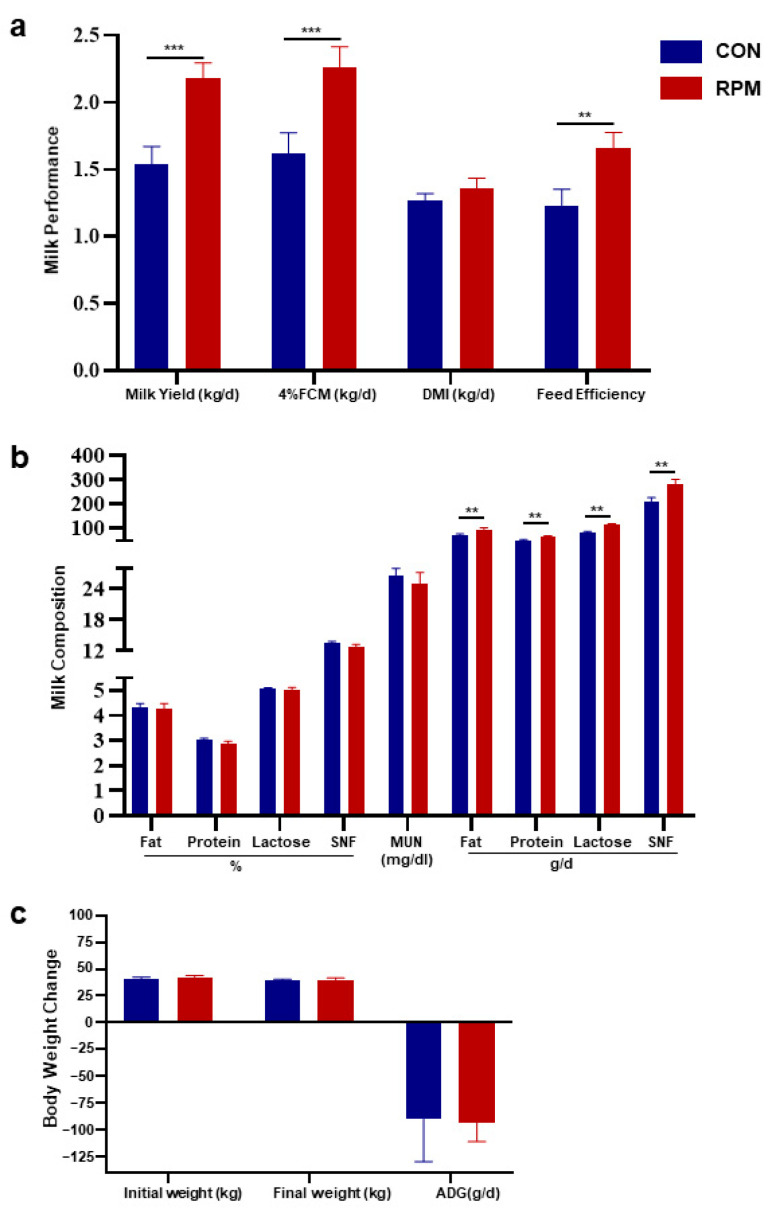
Production performance of dairy goats. (**a**) Lactation performance of dairy goats. (**b**) Milk components and yield of dairy goats. (**c**) Initial body weight, final body weight and ADG of dairy goats. The bars represent the means ± SEs. Significance levels are as follows: ** *p* < 0.01; *** *p* < 0.001.

The RPM group presented significantly increased (*p* < 0.05) milk production, 4% FCM, and feed efficiency (Figure 1). There was no significant difference (*p* > 0.05) in DMI between the two groups. (Figure 1a). With respect to milk composition, no differences (*p* > 0.05) in milk fat, protein, lactose, SNF, or MUN were detected between the two groups. Moreover, the yields of milk fat, protein, lactose, and SNF in the RPM group were significantly greater (Figure 1b, *p* < 0.05). In terms of body weight, no significant differences (*p* > 0.05) in initial body weight, final body weight, or ADG were detected among the dairy goats in the different treatment groups (Figure 1c).

### 3.2. Blood Parameters

**Figure 2:**
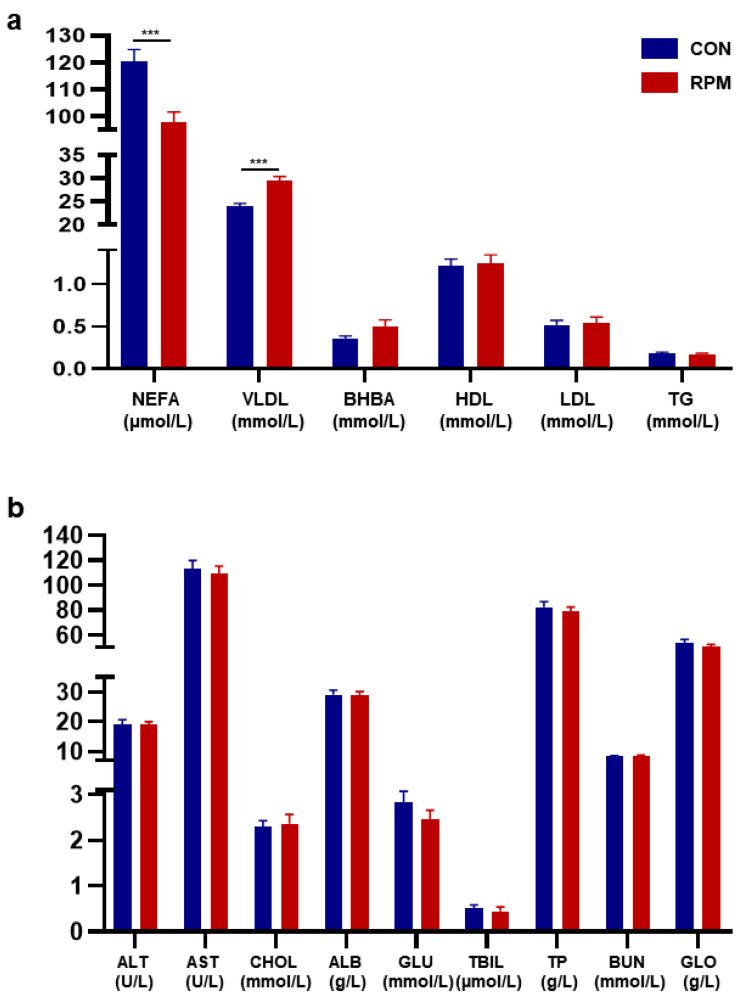
Blood parameters of dairy goats. The bars represent the means ± SEs. (**a**) Concentrations of NEFA, VLDL, BHBA, HDL, LDL and TG in the blood of dairy goats. (**b**) Concentrations of ALT, AST, CHOL, ALB, GLU, TBIL, TP, BUN and GLO in the blood of dairy goats. Significance levels are as follows: *** *p* < 0.001.

Goats fed RPM exhibited significantly lower serum concentrations of NEFA and higher levels of VLDL compared to those fed the control diet (CON) (*p* < 0.05; Figure 2a). However, no significant differences (*p* > 0.05) in BHBA, HDL, LDL, TG, ALT, AST, CHOL, ALB, GLU, TBIL, TP, BUN, or GLO were detected between treatments (Figure 2b).

### 3.3. Fecal Fermentation Parameters

**Figure 3:**
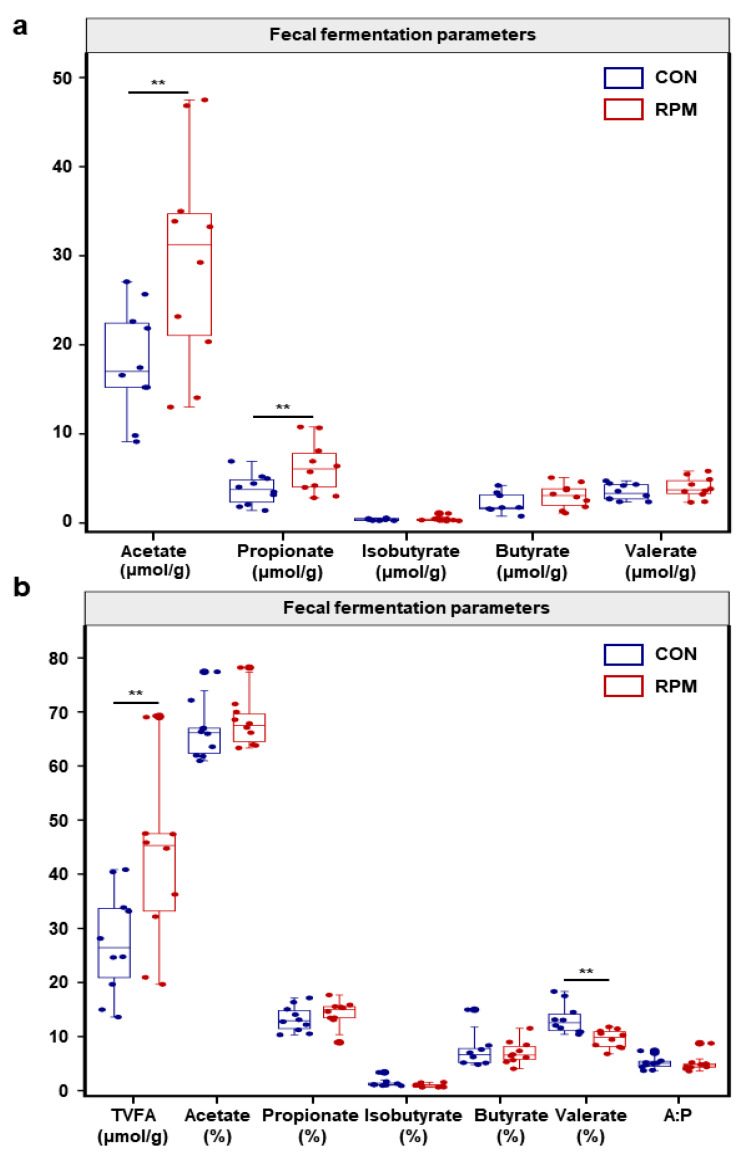
Fecal fermentation of dairy goats. (**a**) Concentrations of acetate, propionate, isobutyrate, butyrate and valerate in the feces of dairy goats. (**b**) Concentrations of TVFAs in the feces of dairy goats, A:P, as well as the percentages of acetate, propionate, isobutyrate, butyrate and valerate. Significance levels are as follows: ** *p* < 0.01.

The fecal VFA analysis (Figure 3) indicated that the concentrations of acetate and propionate (Figure 3a), as well as TVFAs (Figure 3b), in the RPM group were significantly increased (*p* < 0.05), and no differences (*p* > 0.05) were detected in the concentrations of isobutyrate, butyrate, or valerate (Figure 3a). The proportion of valerate (Figure 3b) in the feces of the RPM group was significantly lower (*p* < 0.05), and no differences (*p* > 0.05) in acetate, propionate, isobutyrate, butyrate, or the ratio of acetate to propionate (A:P) were detected between the two groups.

### 3.4. Blood Amino Acid Profile

**Figure 4:**
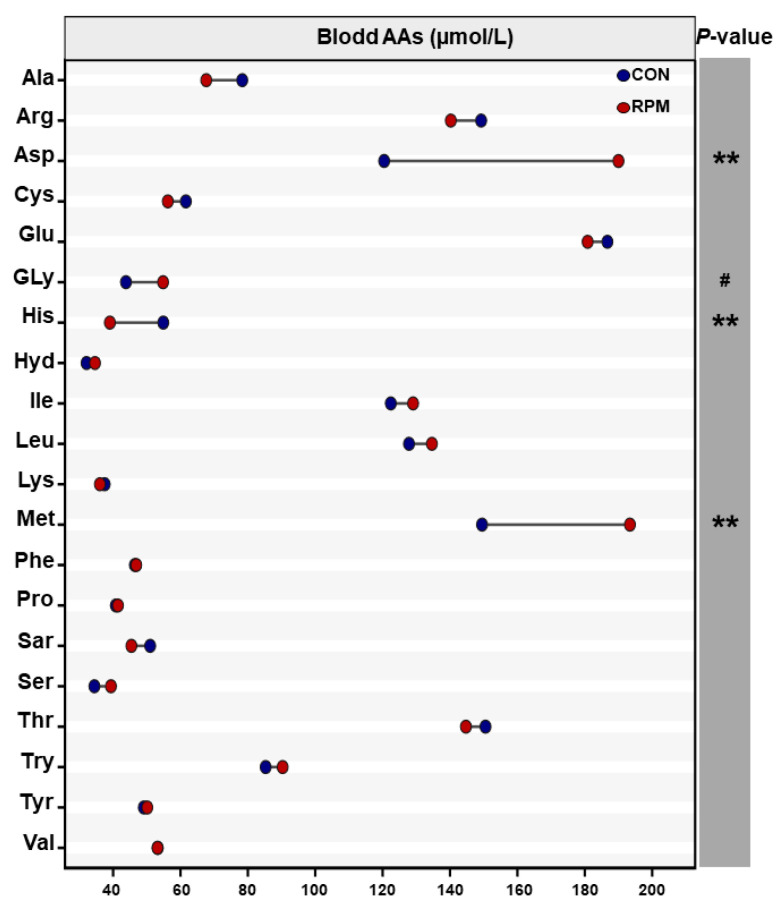
Amino acid profile of dairy goat blood. Significance levels are as follows: ** *p* < 0.01; ^#^ 0.05 < *p* < 0.10.

The concentrations of 20 amino acids were analyzed (Figure 4); among these, the concentrations of aspartic acid (Asp), histidine (His), and methionine (Met) in the blood of the RPM group were significantly greater (*p* < 0.05) than those of the CON group; moreover, glycine (Gly) tended to increase (0.05 ≤ *p* < 0.10). However, no differences (*p* > 0.05) in the concentrations of other amino acids were detected between the two groups.

### 3.5. Fecal Microorganisms

**Figure 5:**
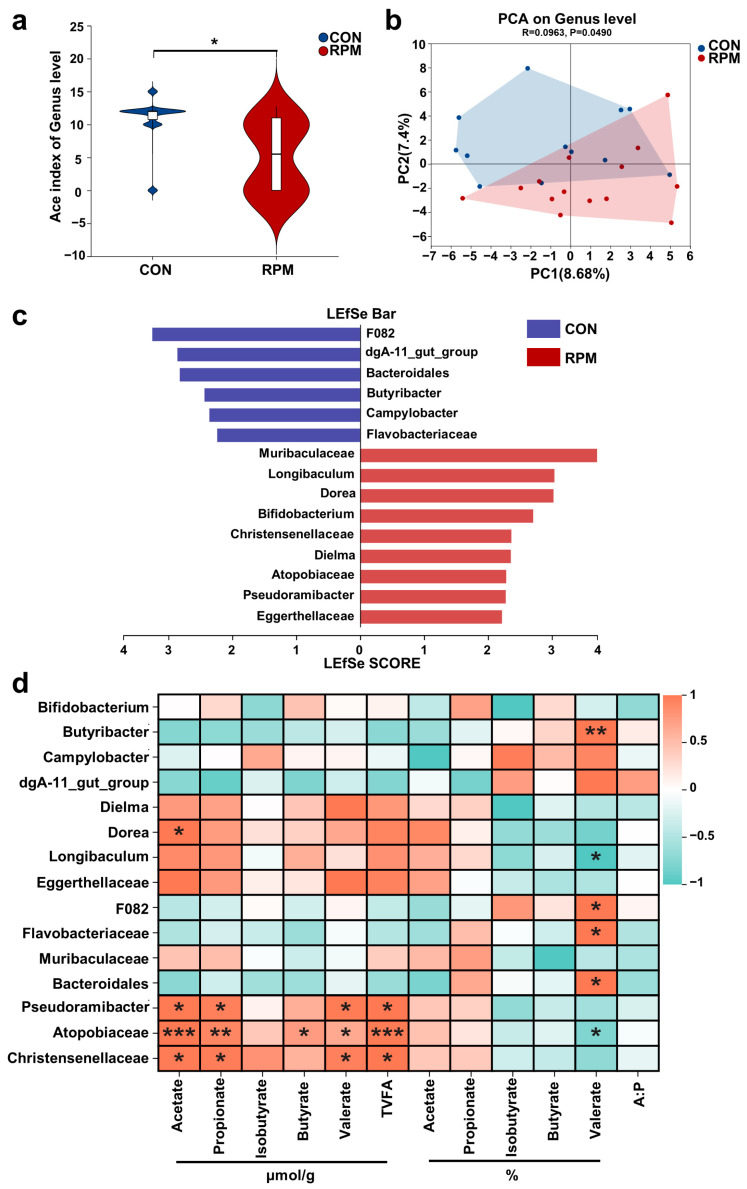
The diversity of the fecal microbiota in dairy goats, the differential bacterial genera among treatments, and their Pearson correlation with VFAs. (**a**) α diversity (Ace index) and (**b**) β diversity (principal component analysis, PCA). (**c**) LEfse analyses of differential microorganisms between the two groups. The LDA discriminant histogram counts the microbial groups that have significant effects (*p* < 0.05, FDA > 2). (**d**) Pearson’s correlation between microbial genus-level differences in bacteria and VFAs. Significance levels are as follows: * *p* < 0.05; ** *p* < 0.01; *** *p* < 0.001.

The α and β diversity of the fecal microbiome, differences in microorganisms at the genus level among treatments, and their correlations with fecal VFAs were further analyzed (Figure 5). The α diversity analysis measured by the Ace index (Figure 5a) and β diversity analysis (Figure 5b) indicated that the richness and diversity of the fecal microbial community in the RPM group were significantly lower (*p* < 0.05) than those in the CON group. LEfSe analysis of the fecal microbiota at the genus level (Figure 5c) revealed that *Muribaculaceae*, *Longibaculum*, *Dorea*, *Bifidobacterium*, *Christensenellaceae*, *Dielma*, *Atopobiaceae*, *Pseudoramibacter*, and *Eggerthellaceae* were significantly enriched in the RPM group, whereas *F082*, *dgA-11 gut group*, *Bacteroidales*, *Butyribacter*, *Campylobacter*, and *Flavobacteriaceae* were significantly enriched in the CON group (FDA > 2, *p* < 0.05).

Pearson correlation analysis was employed to identify associations between differential bacterial genera in feces and VFA phenotypes (Figure 5d). The significantly enriched genera *Dorea*, *Pseudoramibacter*, *Atopobiaceae*, and *Christensenellaceae* in the RPM group were positively correlated with the fecal acetate concentration (r > |0.5|, *p* < 0.05). Furthermore, *Pseudoramibacter*, *Atopobiaceae*, and *Christensenellaceae* in the RPM group were positively correlated with the fecal propionate, valerate, and TVFAs concentrations (r > |0.5|, *p* < 0.05). The *Atopobiaceae* genus, which was significantly enriched in the RPM group, was positively correlated with the concentration of fecal butyrate (r > |0.5|, *p* < 0.05). Additionally, the genera *Butyrobacter*, *F082*, *Flavobacteriaceae*, and *Bacteroidales*, which presented significantly reduced relative abundances in the RPM group, were positively correlated with the percentage of valerate (r > |0.5|, *p* < 0.05). The genera *Longibaculum* and *Atopobiaceae* were negatively correlated with the percentage of valerate (r > |0.5|, *p* < 0.05).

### 3.6. Relationships Between Fecal Microbial Function and VFAs, as Well as Blood Amino Acid Profiles

**Figure 6:**
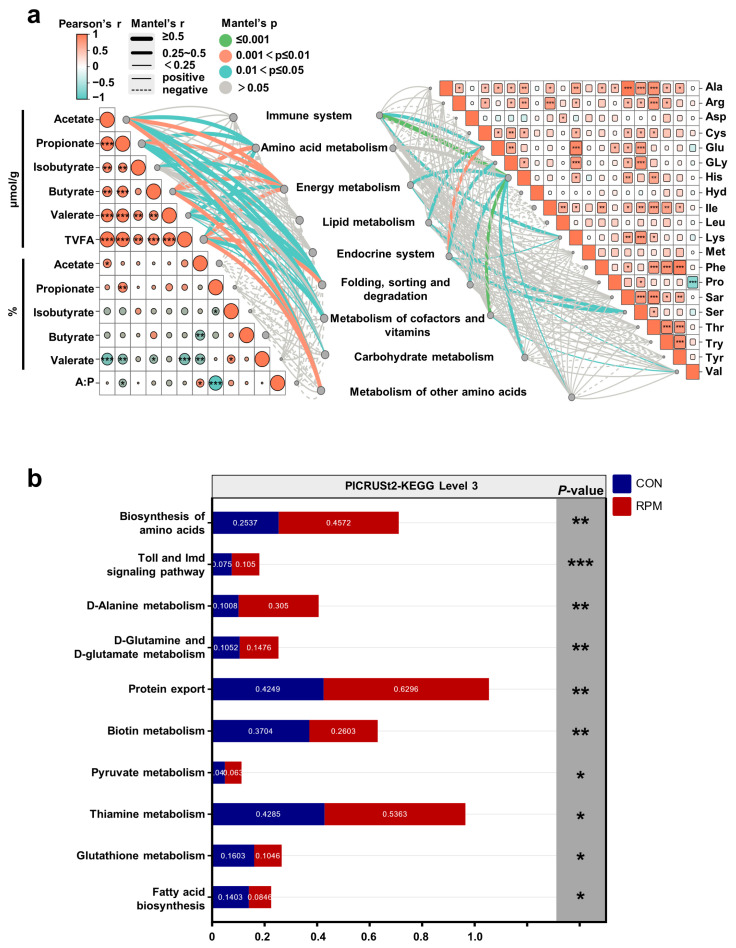
Relationships among microbial function, VFA and the blood amino acid profile. (**a**) Mantel test among microbial function, VFA and the blood amino acid profile matrix. Microbial function analysis was performed via PICRUSt2-KEGG Level 2. VFA and blood amino acid profile comparisons via Pearson’s correlation analysis. The solid lines represent positive correlations, the dotted lines represent negative correlations, and the thickness of the lines represents the size of the correlation coefficient. The color of the line represents the size of the Mantel’s *p* value. (**b**) PICRUSt2-KEGG pathway Level 3 (*p* < 0.05). Significance levels are as follows: * *p* < 0.05; ** *p* < 0.01; *** *p* < 0.001.

To further confirm the interaction among the fecal differential microbiota, VFA, and blood amino acid profiles, we employed the Mantel test to link the relationship matrix of VFA and blood amino acid profiles to the KEGG pathways (Level 2) of the differential bacterial genera (Figure 6a). The concentrations of VFAs (acetate, propionate, isobutyrate, butyrate, valerate, and TVFAs) were significantly positively correlated with the KEGG pathways related to the immune system, amino acid metabolism, energy metabolism, lipid metabolism, the endocrine system, folding, sorting, degradation, metabolism of cofactors and vitamins, carbohydrate metabolism, and metabolism of other amino acids (r > |0.5|, *p* < 0.05). The KEGG pathways related to the immune system, energy metabolism, lipid metabolism, the endocrine system, folding, the metabolism of cofactors and vitamins, carbohydrate metabolism, and the metabolism of other amino acids were significantly positively correlated with the blood amino acid profile (r > |0.5|, *p* < 0.05).

Furthermore, the KEGG pathway (level 3) analysis (Figure 6b) indicated that the biosynthesis of amino acids, the Toll and Imd signaling pathways, D-alanine metabolism, D-glutamine and D-glutamate metabolism, protein export, pyruvate metabolism, and thiamine metabolism were significantly enriched in the RPM group (*p* < 0.05). However, the KEGG pathways related to biotin metabolism, glutathione metabolism, and fatty acid biosynthesis were significantly enriched in the CON group (*p* < 0.05).

## 4. Discussion

This study utilized dairy goats in the early lactation period as research subjects to investigate the effects of dietary supplementation with RPM on the production performance, metabolism, and postintestinal microbiota of these animals. These findings offer a new perspective for the precise breeding and management of dairy goats.

Supplementing the diet of ruminants with RPM is a well-recognized and effective strategy for increasing the supply of metabolizable amino acids, which can reduce the incidence of diseases, increase production performance, and improve liver health [41,42,43]. In the present study, as expected, dietary supplementation with RPM significantly increased milk yield and 4% fat-corrected milk (4% FCM) production in dairy goats. A previous study by Cannas et al. [44] reported that in mid-lactation Salda dairy ewes, milk yield increased by 17% when fed a high-protein diet; however, further increasing dietary crude protein to 18.7% resulted in a decline in milk production [37]. These findings are consistent with those of previous studies [23,45], indicating that supplementation with RPM in the diet of dairy goats can increase milk production. Furthermore, as indicated by Sauvant et al. [46] and Noziere et al. [47], the quantity of essential amino acids required for milk production in the intestine (primarily Met and Lys) is limited, which may lead to a decrease in milk output. Therefore, the increase in the availability of essential amino acids for milk production may account for the observed increase in milk production in this study.

Protein and its amino acid composition are limiting nutrients for milk production in ruminants [48]. Methionine is considered a key limiting amino acid involved in milk synthesis in ruminants [49]. Amino acids in the diet are rapidly degraded by microbes in the rumen, and the amino acids from microbial proteins reaching the small intestine are often insufficient to support higher performance [50]. Therefore, supplementing ruminant diets with methionine can improve animal production performance by influencing feed conversion efficiency as well as the quantity and quality of milk [51,52]. This study revealed that milk fat, protein, lactose, and solid nonfat (SNF) yields all increase significantly. Similarly, in dairy cows, RPM sourced from various sources (13.4–22.1 g/d) is utilized to increase milk yield and significantly increase the yield of milk fat and protein [14,20,21,22]. Moreover, when dairy goats are supplemented with 2.5 g/d RPM during the mid-lactation period, milk production, milk fat, and protein content increase [23]. In contrast, Antongiovanni et al. [53] reported that 5 g/d RPM and 19.0% CP in the diet, as well as Tsiplakou et al. [54] with 6 g/d RPM and 14.0% CP in the diet, had no effect on the milk production and composition of Massese and Chios dairy ewes. In conclusion, the observed differences in the responses of dairy goats to RPM supplementation may be attributed to several factors, including the lactation period, the levels of protein and essential amino acids in the diet, the source, dosage, and duration of RPM supplementation, the lactation potential of the breeds, and the overall nutritional balance of the diet.

It is generally recommended to minimize the extent of negative energy balance during the early postpartum period to improve the success with which young animals adapt to lactation [55]. According to the blood parameter results, supplementation with RPM in the diet of dairy goats significantly reduced the concentration of NEFA in the blood and increased the concentration of VLDL. These findings reflect the beneficial effects of RPM supplementation in dairy goats on liver function and lipid metabolism. During the perinatal period, the breakdown of fat produces NEFA to counteract the adverse effects associated with the initiation of lactation and energy shortages following delivery. Some NEFAs are oxidized to provide energy, and some combine with VLDL and are transported out of the liver to supply substrates for milk fat synthesis, whereas some are re-esterified into TG in the liver [6,7,14]. When fat breakdown exceeds the compensatory capacity of the liver, TGs accumulate in the liver, increasing the risk of fatty liver disease. Studies have shown that NEFA and VLDL in the blood serve as markers of lipid metabolism in perinatal dairy cows [14]. In the present study, the reduction in circulating NEFA in goats fed the RPM diet suggests that RPM supplementation alleviated peripheral lipolysis and energy deficit, likely reflecting enhanced metabolic homeostasis and reduced reliance on adipose tissue mobilization. Concurrently, the increased concentration of VLDL indicates that hepatic TG synthesis and export were promoted, which is consistent with the well-established role of Met as a methyl donor in improving lipid metabolism [56,57].

Studies have shown that hepatic lipid infiltration occurs in periparturient dairy cows fed diets deficient in Met [58,59]. A potential strategy to mitigate fatty liver is dietary supplementation with methyl donors. Met and choline, both recognized as methyl donors, have received considerable attention in this context. However, the efficacy of RPM in alleviating hepatic lipid accumulation has been inconsistent. Dietary supplementation with Met has been reported to reduce hepatic TG concentrations [19]. Another study failed to observe changes in plasma VLDL or liver TG levels [60]. These discrepancies may be attributed to various factors, including the type of methyl donor used, supplementation level, bioavailability, and differences in experimental design. Met serves as a methyl donor may limit PC synthesis by decreasing flux through the phosphatidylethanolamine N-methyltransferase pathway [61]. Because PC is an essential component of the VLDL monolayer [62], limitations in PC synthesis may suppress VLDL assembly [63]. Previous studies have reported that RPM up-regulates the expression of apolipoprotein B100 and microsomal triglyceride transfer protein (MTTP), thereby promoting hepatic VLDL secretion and preventing TG accumulation in the liver [64]. This metabolic adaptation suggests that RPM facilitates the efficient transport of lipids from the liver to peripheral tissues, thereby maintaining energy homeostasis and improving hepatic function during lactation. The alterations in the blood lipid profile observed in this study are highly consistent with the functional enrichment of KEGG pathways related to lipid metabolism and energy metabolism, further supporting the notion that Met supplementation coordinately regulates amino acid and lipid metabolic networks. Overall, the reduced NEFA levels coupled with elevated VLDL concentrations in the RPM-fed goats suggest that RPM-induced improvements in hepatic function and lipid metabolism may represent a key mechanism underlying enhanced production performance in dairy goats.

The blood amino acid profile was assessed two hours after the morning feeding on the basis of dietary treatment. Our results indicated that the levels of Met and Asp in the blood of dairy goats supplemented with RPM significantly increased, whereas His significantly decreased; Gly tended to increase compared with that in CON dairy goats. Elhadi et al. [65] supplemented Lacaune dairy ewes with 5 g/d RPM in the diet and reported a significant increase in the Met concentration in the blood of kids, which is consistent with the results of the present study. The increase in the Met concentration in the blood confirmed that RPM supplementation was effective in dairy goats. Asp is a key substrate in the urea cycle and serves as a precursor for the synthesis of Lys, Thr, Ile, and Met. In the liver, Asp prompts hepatocytes to convert toxic ammonia into urea, thereby improving liver function [66]. In our study, the concentration of Asp in the blood of dairy goats supplemented with RPM significantly increased, which may offer a research perspective on the use of RPM as a functional supplement for improving liver function. Histidine, which is considered a reliable marker of tissue protein mobilization, is released during the catabolism of actin and myosin in skeletal muscle and is not further metabolized within the body [67]. The decrease in His levels in the blood indicated that RPM supplementation led to a lower degree of tissue mobilization, reflecting an improved energy balance within the body.

Furthermore, this study revealed that the levels of Met, Asp, and Gly in the blood of the goats in the RPM group either significantly increased or tended to increase. These amino acids are glucogenic amino acids that can be converted into glucose via hepatic gluconeogenesis to provide energy, playing a crucial role in maintaining internal homeostasis [68]. Additionally, functional prediction of the fecal microbiome revealed that pathways related to amino acid biosynthesis and metabolism were significantly enriched in the RPM group. As mentioned earlier, gut microbes play important roles in amino acid metabolism and synthesis [50]. Therefore, the contribution of fecal microbial amino acid synthesis capacity to the host’s amino acid pool should not be overlooked.

During the perinatal period, reduced feed intake, combined with the increased demand for amino acids necessary to sustain lactation, led to greater tissue protein mobilization. Thus, the overall reduction in circulating amino acids was characteristic of this physiological stage, particularly for limiting amino acids such as Lys and Met. In addition to limiting milk production, the insufficient availability of Met, as described by the amino acid bucket hypothesis, may also hinder the recycling of other amino acids. Consequently, increasing the amount of Met is anticipated to improve the lactation performance of dairy goats by increasing the overall cycling and utilization rates of amino acids.

The colon of dairy goats serves as a fermentation chamber. Its capacity to degrade feed is lower than that of the rumen. The function of the colon is to digest feed that escapes from the rumen and absorbs residual nutrients in the intestinal tract. Reynolds et al. [69] demonstrated that when 1.2 kg/d wheat starch was injected into the true stomach of lactating dairy cows, the pH of the feces decreased from 6.64 to 6.26. This finding revealed that some of the injected starch escapes from the small intestine and is fermented in the colon, with 79% of the energy absorbed by the body as metabolic energy. Therefore, the fecal microbiota (i.e., the colonic microbiota) also has a strong fermentation capacity. Seventy percent of the energy in ruminants is derived from VFAs produced by microbial fermentation. The results of the present study indicate that the concentrations of acetate, propionate, and TVFAs in the feces of dairy goats in the RPM group were significantly increased. These findings indicate that supplementation with RPM in the diet may increase the fermentation capacity of fecal microorganisms for the escape of feed substrates from the rumen and small intestine. Moreover, the metabolites produced by microbial fermentation feed can also serve as supplements to the energy supply of ruminants, thereby improving the production performance of dairy goats. Regrettably, the mechanism by which RPM regulates colon fermentation to supply energy to the body remains unclear at present, and this topic is the focus of our future research.

The colon microbiota is the primary contributor to the production of VFAs. Analyzing the composition and function of the intestinal microbiota is beneficial for further evaluating the effects of RPM on dairy goats. The analysis of fecal microbiota diversity revealed that the α and β diversity in the RPM group was significantly lower than that in the CON group, indicating that the RPM diet led to changes in the composition and structure of the intestinal microbiota. Similarly, Chen et al. [70] demonstrated the important role of RPM in altering the microbiota.

This study found that supplementation with RPM significantly reshaped the composition of the fecal microbiota. In the RPM group, specific bacterial taxa (including *Muribaculaceae, Bifidobacterium, Dorea, Pseudoramibacter, Atopobiaceae*, and *Christensenellaceae*) were markedly enriched and exhibited strong positive correlations with fecal VFA concentrations. Mantel test analyses further revealed that alterations in microbial community structure were closely associated with host amino acid metabolism as well as KEGG pathways related to energy metabolism, lipid metabolism, and immune function.

Furthermore, our research revealed that dietary supplementation with RPM increased the relative abundances of *Muribaculaceae*, *Longibaculum*, *Dorea*, *Bifidobacterium*, *Christensenellaceae*, *Dielma*, *Atopobiaceae*, *Pseudoramibacter*, and *Eggerthellaceae*. *Muribaculaceae* encodes numerous enzymes that degrade carbohydrates, including β-glucosidase, α-arabinase, and α-fucosidase [71,72]. Smith et al. [73] reported that *Muribaculaceae* ferment fibres to produce propionic acid, whereas Ormerod et al. [74] reported that *Muribaculaceae* generates acetic acid, propionic acid, and succinic acid and regulates intestinal barrier function and the immune response. *Muribaculaceae* is considered a promising next-generation probiotic. These results indicate that *Muribaculaceae* can produce VFAs by metabolizing dietary fibre. *Muribaculaceae* can also synthesize B vitamins, including vitamin B1 (thiamine), vitamin B2 (riboflavin), and vitamin B3 (niacin) [75]. With respect to amino acid synthesis, metagenomics predicts that *Muribaculaceae* can synthesize Asp, Gly, and Met [76]. In this study, elevated levels of Asp, Met, and Gly in the blood, along with significant enrichment of KEGG pathways related to thiamine metabolism and amino acid biosynthesis, further confirmed the role of *Muribaculaceae* in vitamin and amino acid synthesis. *Bifidobacterium* can utilize oligosaccharides [77], whereas *Muribaculaceae* breaks down polysaccharides into oligosaccharides [75], which can then be utilized by *Bifidobacterium* to promote its growth and reproduction. Thus, there is cross-feeding or cooperative symbiosis between *Muribaculaceae* and *Bifidobacterium*. This study also revealed that *Bifidobacterium* was significantly enriched in the RPM group. This observation may be attributed to the cross-feeding mechanism between *Muribaculaceae* and *Bifidobacterium*, which contributed to the enrichment of both genera in the RPM group.

The abundance of *Longibaculum* is significantly increased in high-protein diets and is positively correlated with amino acid metabolites and blood glucose concentrations [78]. In this study, *Longibaculum* was significantly enriched in the RPM group, and the energy and amino acid metabolism pathways within the KEGG pathways of this group were also significantly enriched. Thus, after RPM is fed to milk goats, the abundance of *Longibaculum* in the intestinal tract increases, potentially promoting amino acid and energy metabolism. Additionally, *Dorea* [79], *Christensenellaceae* [80], *Atopobiaceae* [81], and *Pseudoramibacter* [82] have been reported to play roles in VFA generation and the regulation of lipid metabolism.

At the metabolic level, fecal concentrations of acetate and propionate were elevated in the RPM group. Acetate and propionate serve as the primary precursors for milk fat and glucose synthesis, respectively. The positive associations between VFA and KEGG pathways related to lipid metabolism, energy metabolism, and the endocrine system provide mechanistic evidence underlying the improved milk yield and feed efficiency observed in RPM-fed dairy goats. Propionate supports hepatic gluconeogenesis and lactose synthesis, whereas acetate contributes to de novo fatty acid synthesis in the mammary gland. Therefore, the increased VFA production under RPM supplementation likely optimizes nutrient partitioning toward milk components, consistent with previous findings that methionine supplementation enhances lactation performance [57,60]. The Mantel test further revealed that microbial KEGG pathways associated with the immune, endocrine, and metabolic systems were significantly correlated with both fecal VFA profiles and blood amino acid patterns. This integrative evidence highlights a coordinated microbe–host metabolic network, in which RPM supplementation not only supplies amino acids to support microbial growth but also enhances amino acid availability for host protein synthesis and lipid metabolism. Collectively, these findings indicate that RPM supplementation enhances VFA production and amino acid biosynthesis by reshaping the colonic microbial metabolic network, thereby improving nutrient utilization efficiency and lactation performance.

## 5. Conclusions

In conclusion, these findings demonstrate that dietary RPM supplementation enhances hepatic lipid metabolism, promotes beneficial gut microbial communities, and stimulates microbial functions related to energy and amino acid metabolism. By improving colon fermentation and overall metabolic efficiency, RPM supplementation ultimately boosts milk production in lactating dairy goats. This work provides new mechanistic insight and practical evidence supporting methionine optimization as a strategy to improve ruminant productivity and health.

## Figures and Tables

**Table 1:** Ingredients and nutrient composition (% of DM) of the basal diet ^1^.

Items	Basal Diets
Ingredient	
Alfalfa hay	33.33
Oat hay	26.67
Ground corn	20.96
Soybean meal	7.88
Wheat bran	6.16
Soybean flour	2.20
Premix ^2^	2.20
NaCl	0.16
NaHCO_3_	0.44
Nutrient	
Starch	15.96
CP	16.71
NDF	40.06
ADF	27.23
Metabolizable AA ^3^	
Arg	6.18
His	2.45
Ile	4.79
Leu	8.06
Lys	6.10
Met	1.65
Phe	4.99
Thr	4.66
Try	1.64
Val	5.50

^1^ Nutrient composition was calculated from wet chemistry analysis of individual feed ingredients sampled weekly throughout the study. Throughout the entire research process, all the treated goats received the same basal diet. ^2^ Provides per kilogram of basal diet: 14.96 mg of Cu, 39.60 mg of Mn, 66.00 mg of Zn, 0.88 mg of Co, 4840.00 IU of vitamin A, 1584.00 IU of vitamin D3, and 66.00 IU of vitamin E. ^3^ Metabolizable AAs were calculated and estimated via Cornell-Penn-Miner Dairy (CPM-Dairy, version 3.0.8.1) software.

## Data Availability

The authors confirm that all data underlying the findings are fully available without restriction.
